# Supplementary material for: HIVprotI: an integrated web based platform for prediction and design of HIV proteins inhibitors
Source: J Cheminform. 2018 Mar 9;10:12. doi: 10.1186/s13321-018-0266-y (PMC5845081; doi:10.1186/s13321-018-0266-y)
Supplement: Supplementary file 2 — Additional file 2. Source code of HIVProtI web server. [file 13321_2018_266_MOESM2_ESM.zip › HIVprotI_Source-code/hivprotI/weka-3-6-6/doc/weka/core/Jython.html]

Jython


---


|  |  |  |
| --- | --- | --- |
| **Overview** \| **Package** \| **Class** \| **Tree** \| **Deprecated** \| **Index** \| **Help** \| **Weka's home** | |  |
| **PREV CLASS**   **NEXT CLASS** | **FRAMES**    **NO FRAMES**     **All Classes** |  |
| SUMMARY: NESTED \| FIELD \| CONSTR \| METHOD | DETAIL: FIELD \| CONSTR \| METHOD |  |


---


## weka.core Class Jython

```
java.lang.Object
  weka.core.Jython
```

**All Implemented Interfaces:**: java.io.Serializable, RevisionHandler

---

``` public class Jython extends java.lang.Object implements java.io.Serializable, RevisionHandler ```

A helper class for Jython.

**Version:**
:   $Revision: 1.2 $

**Author:**
:   fracpete (fracpete at waikato dot ac dot nz)

**See Also:**: Serialized Form

---

| **Field Summary** | |
| --- | --- |
| `static java.lang.String` | `CLASS_PYTHONINERPRETER`             the classname of the Python interpreter |
| `static java.lang.String` | `CLASS_PYTHONOBJECTINPUTSTREAM`             the classname of the Python ObjectInputStream |


**Constructor Summary**

`Jython()`
  
          default constructor, tries to instantiate a Python Interpreter


| **Method Summary** | |
| --- | --- |
| `static java.lang.Object` | `deserialize(java.io.InputStream in)`             deserializes the Python Object from the stream |
| `java.lang.Object` | `getInterpreter()`             returns the currently used Python Interpreter |
| `java.lang.String` | `getRevision()`             Returns the revision string. |
| `static java.lang.Object` | `invoke(java.lang.Object o, java.lang.String methodName, java.lang.Class[] paramClasses, java.lang.Object[] paramValues)`             executes the specified method and returns the result, if any |
| `java.lang.Object` | `invoke(java.lang.String methodName, java.lang.Class[] paramClasses, java.lang.Object[] paramValues)`             executes the specified method on the current interpreter and returns the result, if any |
| `static boolean` | `isPresent()`             returns whether the Jython classes are present or not, i.e. |
| `static void` | `main(java.lang.String[] args)`             If no arguments are given, it just prints the presence of the Jython classes, otherwise it expects a Jython filename to execute. |
| `static java.lang.Object` | `newInstance(java.io.File file, java.lang.Class template)`             loads the module and returns a new instance of it as instance of the provided Java class template. |
| `static java.lang.Object` | `newInstance(java.io.File file, java.lang.Class template, java.io.File[] paths)`             loads the module and returns a new instance of it as instance of the provided Java class template. |
| `static java.lang.Object` | `newInterpreter()`             initializes and returns a Python Interpreter |

**Methods inherited from class java.lang.Object**

`equals, getClass, hashCode, notify, notifyAll, toString, wait, wait, wait`

**Field Detail**

### CLASS\_PYTHONINERPRETER

```
public static final java.lang.String CLASS_PYTHONINERPRETER
```

:   the classname of the Python interpreter

    **See Also:**: Constant Field Values

---


### CLASS\_PYTHONOBJECTINPUTSTREAM

```
public static final java.lang.String CLASS_PYTHONOBJECTINPUTSTREAM
```

:   the classname of the Python ObjectInputStream

    **See Also:**: Constant Field Values


**Constructor Detail**

### Jython

```
public Jython()
```

:   default constructor, tries to instantiate a Python Interpreter


**Method Detail**

### getInterpreter

```
public java.lang.Object getInterpreter()
```

:   returns the currently used Python Interpreter

    :   **Returns:**: the interpreter, can be null

---


### invoke

```
public java.lang.Object invoke(java.lang.String methodName,
                               java.lang.Class[] paramClasses,
                               java.lang.Object[] paramValues)
```

:   executes the specified method on the current interpreter and returns the
    result, if any

    :   **Parameters:**: `o` - the object the method should be called from, e.g., a Python Interpreter: `methodName` - the name of the method: `paramClasses` - the classes of the parameters: `paramValues` - the values of the parameters **Returns:**: the return value of the method, if any (in that case null)

---


### isPresent

```
public static boolean isPresent()
```

:   returns whether the Jython classes are present or not, i.e. whether the
    classes are in the classpath or not

    :   **Returns:**: whether the Jython classes are available

---


### newInterpreter

```
public static java.lang.Object newInterpreter()
```

:   initializes and returns a Python Interpreter

    :   **Returns:**: the interpreter or null if Jython classes not present

---


### newInstance

```
public static java.lang.Object newInstance(java.io.File file,
                                           java.lang.Class template)
```

:   loads the module and returns a new instance of it as instance of the
    provided Java class template.

    :   **Parameters:**: `filename` - the path to the Jython module, incl. filename: `template` - the template for the returned Java object **Returns:**: the Jython object

---


### newInstance

```
public static java.lang.Object newInstance(java.io.File file,
                                           java.lang.Class template,
                                           java.io.File[] paths)
```

:   loads the module and returns a new instance of it as instance of the
    provided Java class template. The paths are added to 'sys.path' - can
    be used if the module depends on other Jython modules.

    :   **Parameters:**: `filename` - the path to the Jython module, incl. filename: `template` - the template for the returned Java object: `paths` - additional paths to add to "sys.path" **Returns:**: the Jython object

---


### invoke

```
public static java.lang.Object invoke(java.lang.Object o,
                                      java.lang.String methodName,
                                      java.lang.Class[] paramClasses,
                                      java.lang.Object[] paramValues)
```

:   executes the specified method and returns the result, if any

    :   **Parameters:**: `o` - the object the method should be called from, e.g., a Python Interpreter: `methodName` - the name of the method: `paramClasses` - the classes of the parameters: `paramValues` - the values of the parameters **Returns:**: the return value of the method, if any (in that case null)

---


### deserialize

```
public static java.lang.Object deserialize(java.io.InputStream in)
```

:   deserializes the Python Object from the stream

    :   **Parameters:**: `in` - the stream to use **Returns:**: the deserialized object

---


### getRevision

```
public java.lang.String getRevision()
```

:   Returns the revision string.

    :   **Specified by:**: `getRevision` in interface `RevisionHandler`
    :   **Returns:**: the revision

---


### main

```
public static void main(java.lang.String[] args)
```

:   If no arguments are given, it just prints the presence of the Jython
    classes, otherwise it expects a Jython filename to execute.

    :   **Parameters:**: `args` - commandline arguments


---


|  |  |  |
| --- | --- | --- |
| **Overview** \| **Package** \| **Class** \| **Tree** \| **Deprecated** \| **Index** \| **Help** \| **Weka's home** | |  |
| **PREV CLASS**   **NEXT CLASS** | **FRAMES**    **NO FRAMES**     **All Classes** |  |
| SUMMARY: NESTED \| FIELD \| CONSTR \| METHOD | DETAIL: FIELD \| CONSTR \| METHOD |  |


---
